# A Δ38 Deletion Variant of Human Transketolase as a Model of Transketolase-Like Protein 1 Exhibits No Enzymatic Activity

**DOI:** 10.1371/journal.pone.0048321

**Published:** 2012-10-31

**Authors:** Stefan Schneider, Stefan Lüdtke, Kathrin Schröder-Tittmann, Cindy Wechsler, Danilo Meyer, Kai Tittmann

**Affiliations:** Albrecht-von-Haller-Institute and Göttingen Center for Molecular Biosciences, Department of Bioanalytics, Georg-August-University Göttingen, Germany; Deutsches Krebsforschungszentrum, Germany

## Abstract

Besides transketolase (TKT), a thiamin-dependent enzyme of the pentose phosphate pathway, the human genome encodes for two closely related transketolase-like proteins, which share a high sequence identity with TKT. Transketolase-like protein 1 (TKTL1) has been implicated in cancerogenesis as its cellular expression levels were reported to directly correlate with invasion efficiency of cancer cells and patient mortality. It has been proposed that TKTL1 exerts its function by catalyzing an unusual enzymatic reaction, a hypothesis that has been the subject of recent controversy. The most striking difference between TKTL1 and TKT is a deletion of 38 consecutive amino acids in the N-terminal domain of the former, which constitute part of the active site in authentic TKT. Our structural and sequence analysis suggested that TKTL1 might not possess transketolase activity. In order to test this hypothesis in the absence of a recombinant expression system for TKTL1 and resilient data on its biochemical properties, we have engineered and biochemically characterized a “pseudo-TKTL1” Δ38 deletion variant of human TKT (TKTΔ38) as a viable model of TKTL1. Although the isolated protein is properly folded under *in vitro* conditions, both thermal stability as well as stability of the TKT-specific homodimeric assembly are markedly reduced. Circular dichroism and NMR spectroscopic analysis further indicates that TKTΔ38 is unable to bind the thiamin cofactor in a specific manner, even at superphysiological concentrations. No transketolase activity of TKTΔ38 can be detected for conversion of physiological sugar substrates thus arguing against an intrinsically encoded enzymatic function of TKTL1 in tumor cell metabolism.

## Introduction

Transketolase (TKT, EC 2.2.1.1) is a ubiquitous thiamin diphosphate (ThDP) and Me^2+^-dependent enzyme that catalyzes the reversible transfer of two-carbon ketol units between ketose and aldose phosphates in the nonoxidative part of the pentose phosphate pathway (PPP) 1,2,3. TKT thus provides, along with transaldolase (TAL), which transfers three-carbon units, a reversible connection between glycolysis and the PPP. The major metabolic purposes of the PPP are to generate reducing equivalents in the form of NADPH, and to supply ribose 5-phosphate for nucleotide biosynthesis and erythrose 4-phosphate for biosynthesis of aromatic amino acids.

Human TKT has attracted considerable attention as it was implicated in several pathological disorders such as the Wernicke-Korsakoff Syndrome, Alzheimer’s disease, diabetes and cancer [4], [5], [6]. In fact, the vast majority of ribose for nucleic acid biosynthesis in cancer cells is provided by the non-oxidative part of the PPP through activity of TKT and TAL. There is experimental evidence that TKT activity can be effectively inhibited by applying coenzymatically inactive thiamin analogs, which in some but not all instances reduced the proliferation of tumor cells [7], [8], [9], [10].

Besides authentic TKT, the human genome encodes for two related proteins, termed transketolase-like proteins 1 and 2 (TKTL1 and TKTL2) [11]. While a possible biological function of human TKTL2 remains to be discovered, TKTL1 has been suggested to be a critical determinant for energy metabolism, growth and invasion efficiency of malignant tumors [12]. In several studies, a direct correlation between the cellular expression levels of TKTL1 and patient prognosis (mortality) was reported [13], [14], [15]. It has been hypothesized that TKTL1 could exhibit enzymatic activity that allows ATP synthesis in hypoxic and cancer cells in a pathway analogous to the phosphoketolase pathway present in some heterofermentative bacteria [12]. However, the presumed key role of TKTL1 in cancer cell metabolism has been put into question in numerous recent studies, where no overexpression of TKTL1 could be detected in several malignant tumor cell lines [16], [17].

**Figure 1:**
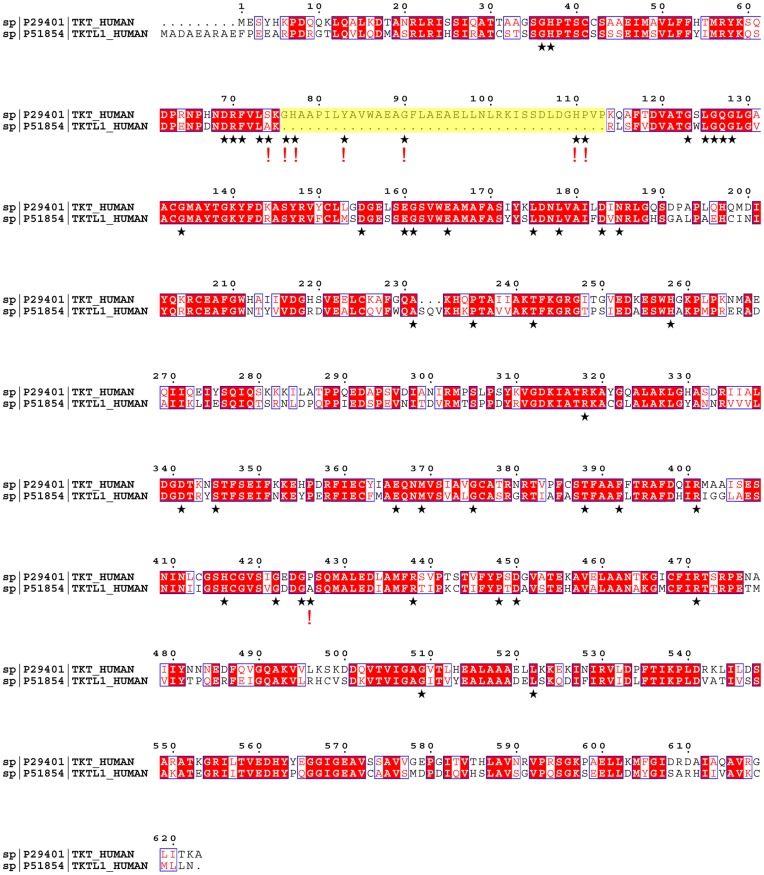
Sequence alignment of human TKT versus human TKTL1 (identifier P51854-3) using programs ClustalW2 and ESPript 2.2. Numbering corresponds to the sequence of human TKT. Identical residues are indicated by a red background, similar residues by red characters. The major difference in sequence between the two proteins, a deletion of 38 amino acid residues in the N-terminal part, is highlighted in yellow. Residues, which are invariant amongst all TKT sequences, are indicated by an asterisk. Positions in the TKTL1 sequence, where an invariant residue is missing or replaced, are highlighted by an exclamation mark.

**Figure 2:**
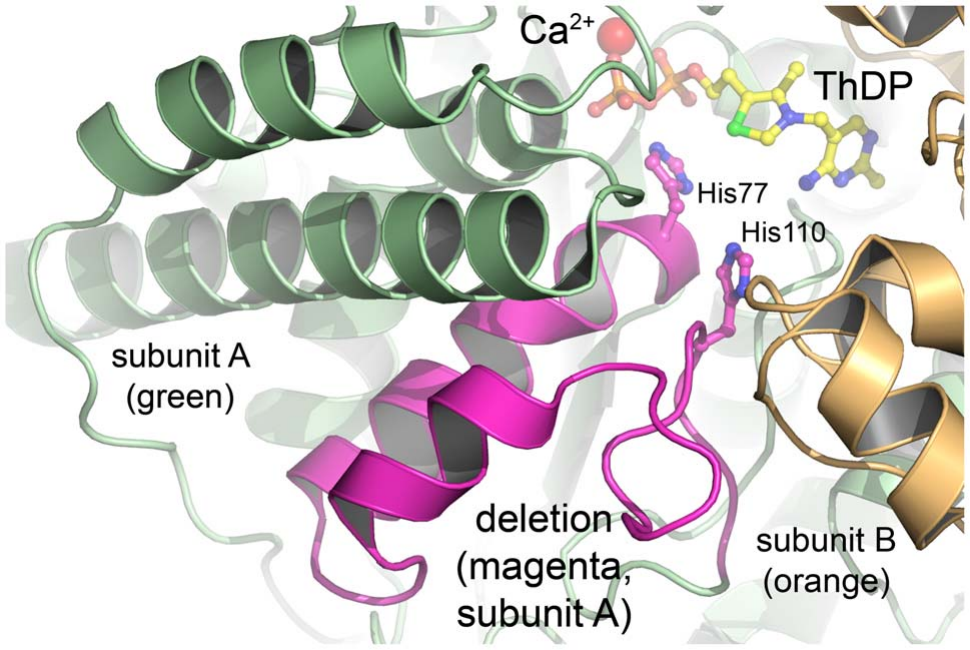
Active site structure of human TKT highlighting the sequence, which has been deleted in TKTΔ38 to generate a viable model of TKTL1. The structure of human TKT (pdb code 3 mos) with bound cofactors ThDP (yellow sticks) and Ca^2+^ (red sphere) is shown in cartoon representation. The individual subunits of the functional dimer are shown in green (subunit A) and orange (subunit B), respectively. The deleted sequence comprising residues Gly76-Pro113 of subunit A is colored in magenta. Two His residues of this sequence, which are supposedly critical for cofactor binding and enzymatic activity, are shown in stick representation.

TKTL1 (canonical sequence P51854-3) exhibits a very high sequence identity of 61% and sequence similarity of 75% with TKT (Fig. 1). The major difference in sequence between TKTL1 and TKT is a deletion of 38 consecutive amino acids in the N-terminal PP (pyrophosphate) domain of TKTL1 comprising the equivalent residues 76–113 in TKT. Most notably, this sequence contains numerous residues (His77, Tyr83, Gly90, His110, Pro111), which are totally invariant amongst all transketolase sequences [18]. In total, TKTL1 lacks 8 invariant TKT residues (Fig. 1) (18). Our previous structural analysis of human TKT by X-ray crystallography revealed that the 38 mer sequence in question folds into a loop–helix-turn-helix–loop motif that constitutes part of the cofactor binding and active site (Fig. 2) [19]. This structural information and mutagenesis studies on TKTs from different organisms including the human enzyme indicate that several residues of this sequence play critical roles in cofactor binding (His77) and in substrate binding/catalysis (His77, His110) (see Fig. 2) [20], [21], [22]. In addition, several residues line the dimer interface and make intimate contacts with the neighboring subunit suggesting a role of this segment for dimer formation (the homodimer is the biologically active assembly). In view of the available structural data and sequence information, we have hypothesized that TKTL1 might not possess enzymatic TKT activity as it lacks numerous invariant residues required for cofactor binding and catalysis [19].

Over the past years, we have unsuccessfully attempted to establish a recombinant expression system for TKTL1 and to study its biochemical properties *in vitro* (see Methods S1, Fig. S1, S2). These efforts included expression trials in *E. coli* using a synthetic, codon-optimized gene (this approach worked well for expression of authentic TKT, ref *19*) as well as in different insect cell lines (Sf9, Sf21, Hi5). We observed that TKTL1 is not expressed in soluble form, even when fused to proteins that potentially increase the solubility such as e.g. the SUMO protein, but is exclusively found to accumulate in inclusion bodies. In the absence of a functioning expression system for TKTL1 and the resultant lack of reliable biochemical and biophysical data on it, we describe here functional and structural properties of a genetically engineered Δ38 deletion variant of TKT (TKTΔ38) as a minimal model of TKTL1, which lacks residues Gly76-Pro113 (see Fig. 1). In contrast to the aforementioned difficulties to set up an expression system for TKTL1, we could previously establish a robust recombinant expression system for human TKT in *E. coli* that allows for the production of soluble and active material in sufficient amounts to undertake a rigorous biochemical characterization. The TKTΔ38 deletion variant seems to be, in our eyes, a viable model of TKTL1 as both proteins exhibit a sequence identity of 65% and a sequence similarity of 80%.

After recombinant and soluble expression of TKTΔ38 in *E. coli*, we have tested highly purified protein for cofactor binding and enzymatic activity versus full-length TKT under fully standardized conditions. In addition, we have characterized the impact of the deletion on the overall structure and on the stability of the protein. The results obtained on TKTΔ38 are discussed in light of the proposed enzymatic function of TKTL1 in tumor cell metabolism.

## Experimental Procedures

### Generation of the Deletion Construct by Overlap Extension PCR

An overlap extension method was used to create the Δ38 amino acid deletion variant on the genetic level, for which our previously generated codon-optimized human *tkt*-gene (in pET28a expression vector) served as template [19]. Overlap extension was carried out by employing the Phusion™ High Fidelity DNA polymerase system (Finnzymes, Espoo, Finland). The following primers were used:

Deletion forward,

5′–CGTTTTGTTCTGAGCAAAAAACAGGCGTTTACCGATGTG –3′ (sense);

deletion reverse,

5′– CATCGGTAAACGCCTGTTTTTTGCTCAGAACAAAACGATCG –3′ (antisense),

T7 forward,

5′ – TAATACGACTCACTATAGGG –3′ (sense),

T7 reverse,

5′– AGCTAGTTATTGCTCAGCGG –3′ (antisense).

In a first step, the sequences encoding for the two parts of the protein (residues 1–75 and residues 114–623) were amplified individually by using the following primer combinations: T7 forward/deletion reverse and T7 reverse/deletion forward. In a subsequent PCR reaction, the amplicons were fused by usage of T7 forward and T7 reverse primers. The shortened *tkt* construct was cloned into the amplification plasmid pJET (Fermentas) and transferred as *Nco*I/*Xho*I fragment into the pET28a expression vector. Correctness of the introduced deletion was confirmed by complete sequencing of the gene.

### Gene Expression and Purification of Human Transketolase Δ38 Deletion Variant

The pET28a vector encoding for C-terminally His-tagged Δ38 deletion variant of human transketolase was transformed into *E. coli* BL21* cells (Invitrogen) by the method of Inoue *et al.* [23]. Transformed cells were grown on LB/Agar plates containing 35 µg/mL kanamycin over night at 37°C. A single colony was used to inoculate 200 mL of overnight culture (LB medium supplemented with 35 µg/mL kanamycin) in a 1 L Erlenmeyer flask. The overnight culture was centrifuged (6000 rpm, 20 min, 4°C), and cells were resuspended in 100 mL SB medium containing 35 µg/mL kanamycin. This cell suspension was then used to inoculate 400 mL of SB medium containing 35 µg/mL kanamycin and 100 µg/mL thiamin. At an A_600_ of ∼0.6, gene expression was induced by addition of 100 µM IPTG, and cell cultures were grown for 18 h at 12°C and 200 rpm. Cultivation at higher temperatures resulted in formation of insoluble protein as inclusion bodies. Cells were harvested by centrifugation at 6000 g for 20 min at 4°C. Thereafter, harvested cells were shock-frozen in liquid nitrogen and stored at −80°C until usage. Human full-length TKT was expressed using the same protocol.

For protein purification, 20 g of cells were thawed and resuspended on ice in 60 mL of buffer containing 20 mM Tris-HCl pH 7.6, 500 mM NaCl, 20 mM imidazole, 1 mM CaCl_2_ and 100 µM thiamin diphosphate. Moreover, DNase (5 µg/mL), MgCl_2_ (1 mM), lysozyme (0.2 mg/mL) and PMSF (1 mM) were added. The suspension was stirred on ice for 30 min. Cells were disrupted by repeated passages through a microfluidizer (Microfluidics, Newton, MA, US). Cell debris was removed by centrifugation at 54000 g for 30 min at 4°C. The supernatant was loaded onto a Ni^2+^-NTA column (GE Healthcare) previously equilibrated with 10 column volumes of 20 mM Tris-HCl, pH 7.6, 500 mM NaCl, 20 mM imidazole, 1 mM CaCl_2_ and 100 µM thiamin diphosphate. Variant TKTΔ38 was eluted with a linear gradient (0–100%) elution buffer (20 mM Tris-HCl, pH 7.6, 500 mM NaCl and 300 mM imidazole) over a total volume of 20 column volums. Fractions containing TKTΔ38 were pooled and loaded onto a HiPrep™ 26/10 desalting column (GE Healthcare) equilibrated with 50 mM glycyl-glycine, pH 7.6 containing 500 mM NaCl. Finally, the protein concentration was adjusted to 15–25 mg/mL by ultrafiltration using a microconcentrator (VIVASPIN, Sartorius) with a molecular weight cut-off of 50000 at 4°C. Full-length TKT was purified using the identical protocol. Protein homogeneity was judged by SDS-PAGE analysis.

All experiments discussed in this study were conducted with three independent batches of each protein purified from independent cell cultivations.

### Kinetic Analysis of Enzymatic Activity

The enzymatic activity of TKTΔ38 and full-length TKT for conversion of physiological substrates D-xylulose 5-phosphate (X5P, Sigma-Aldrich, purity >90%) and D-ribose 5-phosphate (R5P, Sigma-Aldrich, purity >98%) into products D-glyceraldehyde 3-phosphate (G3P) and D-sedoheptulose 7-phosphate (S7P) was measured in a coupled spectrophotometric assay using the auxiliary enzymes triosephosphate isomerase (TIM) and *sn*-glycerol-3-phosphate : NAD^+^2-oxido-reductase (G3PDH). This assay detects formation of product G3P that derives from TKT-catalyzed cleavage of substrate X5P. The concomitant oxidation of NADH was followed spectrophotometrically at 340 nm in 50 mM glycyl-glycine, pH 7.6 containing 500 mM NaCl at 30°C. The assay contained as final concentrations 220 µM NADH, 3.6 units of TIM/G3PDH enzyme mix, 1 mM ThDP, 1 mM CaCl_2_, 5 mM of X5P and R5P, and varied concentrations of TKTΔ38 and full-length TKT (0.05–2 mg/mL).

### Circular Dichroism Spectroscopy

In order to analyze the secondary structure contents, far-UV circular dichroism (CD) spectra of full-length TKT and TKTΔ38 were recorded in a 1 mm quartz cuvette at 20°C using a Chirascan CD spectrometer (Applied Photophysics) equipped with Peltier temperature control. Spectra were measured at a protein concentration of 0.1 mg/mL in 50 mM sodium phosphate buffer, pH 7.6 containing 2.5 mM MgCl_2_ and 100 µM ThDP. In case of TKTΔ38, the buffer additionally contained 500 mM NaCl to prevent protein aggregation. The scanning range was adjusted to 260–180 nm. The spectra were corrected for buffer contributions and converted to mean residue ellipticity according to Schmid [24]. The secondary structure contents were estimated using program CDNN [25].

Thermal denaturation of both proteins was analyzed at a wavelength of 222 nm within a temperature range of 20–94°C. A heating rate of 1°C/min was adjusted, the data interval was set at 0.5°C at an accumulation time of 12 sec. Spectra were recorded before (at 20°C) and after (94°C and 20°C) thermal denaturation.

As enzyme-bound ThDP in all hitherto spectroscopically characterized TKTs gives rise to a negative CD signal centered around 320 nm [26], [27], we tested TKTΔ38 and full-length TKT for cofactor binding by measuring near-UV CD spectra in a 10 mm quartz cuvette at 20°C. Spectra were recorded at a protein concentration of 2 mg/mL in 50 mM glycyl-glycine buffer, pH 7.6 containing 500 mM NaCl. The scanning range was adjusted to 400–290 nm. The spectra were corrected for buffer contributions.

### Analytical Gel Filtration

Analytical gel filtration experiments were carried out on an ÄKTA Purifier HPLC-system (GE Healthcare) to assess the oligomerization state of TKTΔ38 and full-length TKT. A sample volume of 200 µL (4 mg/mL protein in 50 mM glycyl-glycine buffer, pH 7.6 containing 500 mM NaCl) was loaded on an analytical Superdex 200 10/30 column (GE Healthcare) at 6°C. The apparent molecular masses of TKTΔ38 and full-length TKT were estimated using a set of protein standards (thyroglobulin 670 kDa, γ-globulin 158 kDa, ovalbumin 44 kDa, myoglobin 17 kDa and cobalamine 1.35 kDa) (Biorad).

### NMR Spectroscopy

Acid quench/NMR experiments were carried out to test whether as-isolated TKTΔ38 contains tightly bound ThDP cofactor as previously demonstrated for full-length TKT. 400 µL of TKTΔ38 (7.5 mg/mL, corresponding to 117 µM monomer) in 50 mM glycyl-glycine buffer, pH 7.6 containing 500 mM NaCl were quenched by addition of 200 µL acidic quench solution as previously described [19], [28]. After centrifugation, the precipitated protein was discarded and the supernatant was analyzed by one dimensional ^1^H-NMR spectroscopy using a Bruker Ultrashield 400 MHz NMR spectrometer (Bruker, MA, US). As positive control the same experiment was carried out with full-length TKT.

## Results and Discussion

**Figure 3:**
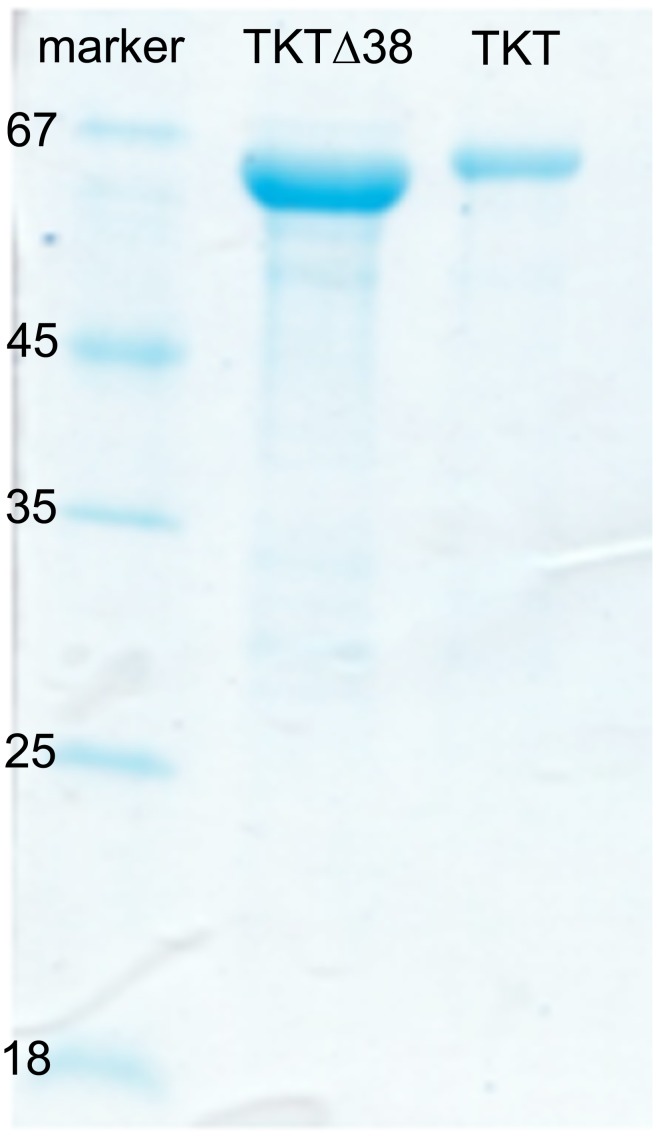
Representative SDS-PAGE analysis of purified TKTΔ38 and full-length TKT. Note the smaller molecular weight of the deletion variant compared to the wild-type form.

Using our previously established recombinant expression system that relies on a synthetic codon-optimized *tkt*-gene for heterologous expression in *E. coli* [19], we could purify sufficient amounts of human full-length TKT and TKTΔ38 to undertake a rigorous comparative biochemical and biophysical characterization of highly purified material (Fig. 3). Starting from ∼5 L cell culture, we could purify ∼15–20 mg of TKTΔ38 and ∼10 mg of full-length TKT to homogeneity.

In initial purification trials, which relied on the protocol established for full-length TKT, we observed that TKTΔ38 is prone to aggregation in low-salt buffer (50 mM glycyl-glycine pH 7.6) typically used to assay TKT. While testing different buffer additives, we found out that an addition of 500 mM NaCl prevents aggregation of TKTΔ38 at ambient temperatures.

### Analysis of Secondary Structure and Thermal Stability by far-UV CD Spectroscopy

The reduced stability of TKTΔ38 compared to full-length TKT in low-salt buffer clearly indicates an impact of the deletion on the thermodynamic stability of the protein. In order to detect potential changes in the secondary structure content, we measured far-UV CD spectra of both proteins.

**Figure 4:**
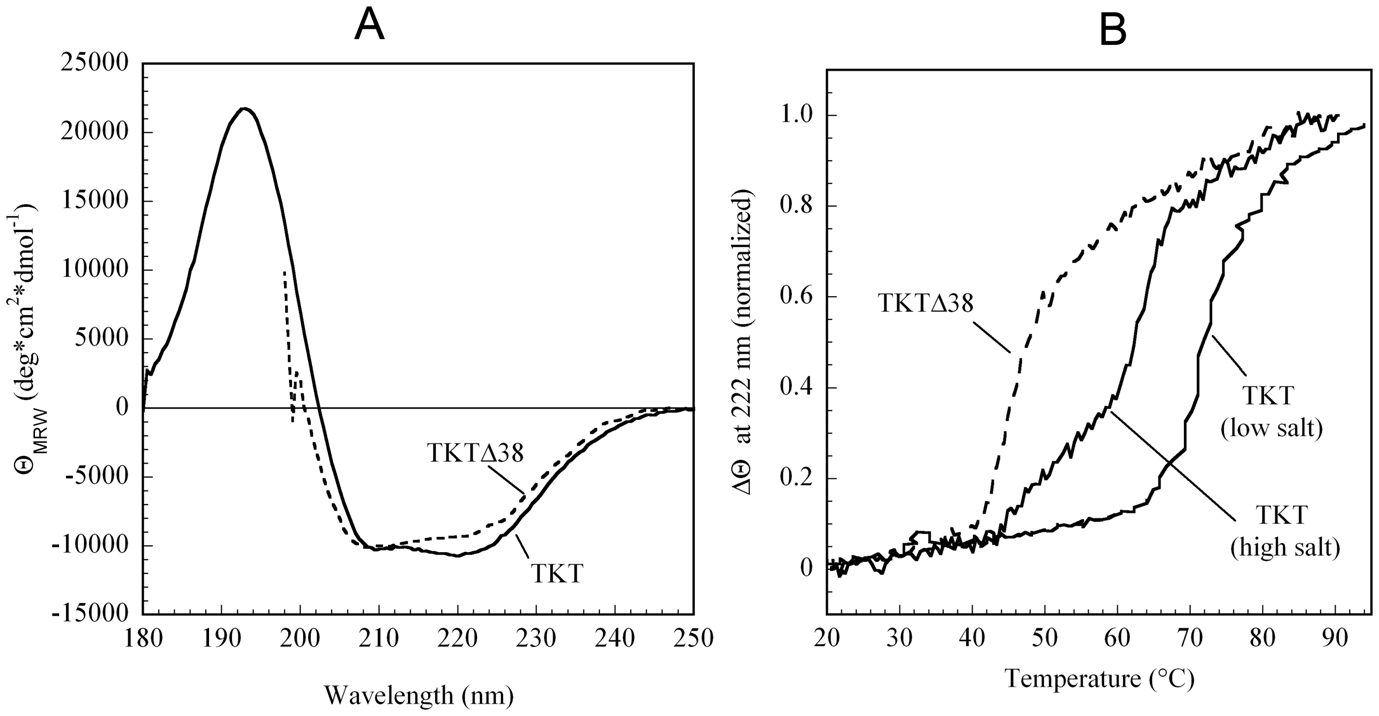
Far-UV CD spectra and thermal unfolding of TKT and variant TKTΔ38. (**A**) CD spectra of full-length TKT and TKTΔ38 were recorded at a protein concentration of 0.1 mg/mL in 50 mM sodium phosphate buffer, pH 7.6 containing 2.5 mM MgCl_2_ and 100 µM ThDP at 20°C. In case of the deletion variant, the buffer additionally contained 500 mM NaCl. (**B**) Thermal unfolding of TKT and TKTΔ38 as detected by the change of the far-UV CD signal at 222 nm. Unfolding was analyzed both in high-salt buffer (50 mM sodium phosphate, pH 7.6, 500 mM NaCl, 2.5 mM MgCl_2_, 100 µM ThDP) and low-salt buffer (same as above but devoid of NaCl). Note that TKTΔ38 is too unstable in low-salt buffer as to allow a spectroscopic analysis under these conditions. Full conditions are detailed in the Experimental Procedures.

Full-length TKT exhibits a far-UV CD spectrum that is typical for an (α/β)-protein and consists of two negative bands centered around 220 nm and 210 nm, and a positive band at 195 nm (Fig. 4a). Using the Cdnn software, we estimated an α-helical content of 35% and a relative fraction of β-sheets of 16%. This estimate is in very good agreement with the secondary structure content observed in the crystal structure of TKT (36% α-helices and 15% β-sheets as calculated by software Promotif, pdb code 3MOS).

The CD spectrum of TKTΔΔ38 in high-salt buffer is limited to wavelengths >200 nm due to the strong absorbance of the salt. Nonetheless, the spectra clearly indicate a similar overall (α/β)-fold of the protein. Completely unfolded segments, which would give a strong negative contribution at around 190 nm in the CD spectrum, are absent for TKTΔ38. Notably, the helicity of TKTΔ38 monitored at 222 nm is slightly smaller compared to full-length TKT. This is, however, an expected finding because the deleted 38 mer sequence contains two short helices (Fig. 2). In line with that qualitative observation, a quantitative analysis of the secondary structure content using program Cdnn yielded a slightly reduced α-helical content of 31% and 17% β-sheets.

Next, we analyzed the thermal unfolding of TKT and TKTΔ38 at 222 nm (Fig. 4b), which turned out to be an irreversible process in both cases. The apparent melting temperature of full-length TKT amounts to ∼72°C in low-salt buffer (50 mM glycyl-glycine, pH 7.6). The thermal stability of TKTΔ38 under these conditions is dramatically reduced. In fact, the variant aggregates almost instantaneously in low-salt buffer at 20°C indicating a difference in the melting temperature of >50°C under these conditions. After addition of 500 mM NaCl, TKTΔ38 is thermally stable at 20°C and unfolding occurs at an apparent melting temperature of ∼44°C. Under identical conditions (high-salt buffer), full-length TKT shows a multiphasic unfolding with a minor transition at 53°C and a major one at 64°C. Taken together, it can be said that the deletion has a strong destabilizing effect on thermal stability of the protein. The super-physiological salt concentrations required to stabilize TKTΔ38 pose the immediate question whether TKTL1 exhibits sufficient thermal stability at 37°C in the cellular context.

### Analysis of Enzymatic Transketolase Activity

**Figure 5:**
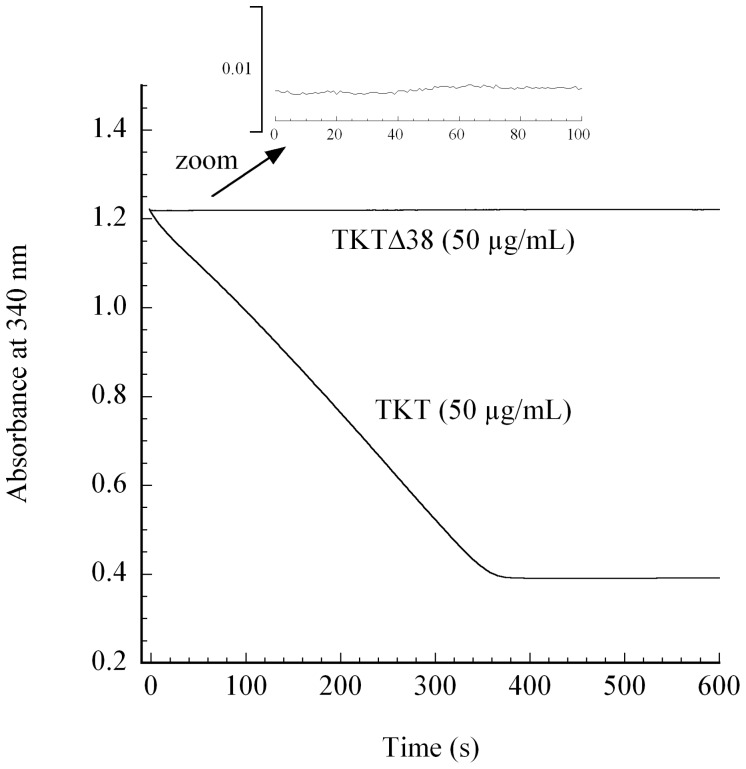
Steady-state kinetic analysis of enzymatic activity of full-length TKT and variant TKTΔ38. Enzymatic activity for conversion of physiological substrates X5P and R5P into products G3P and S7P was analyzed in a coupled spectrophotometric assay at 340 nm (NADH depletion) and 30°C. Full conditions are detailed in the Experimental Procedures. Note that we were unable to detect any enzymatic activity in case of variant TKTΔ38.

Potential enzymatic activity of highly purified TKT and TKTΔ38 was examined in a coupled spectrophotometric assay for conversion of physiological substrates X5P and R5P into products S7P and G3P (Fig. 5). Since TKTΔ38 is unstable in low-salt buffer typically used for the TKT activity assay, all experiments were conducted in the presence of 500 mM NaCl. In the presence of this high salt concentration, full-length TKT exhibits slightly less activity (maximal activity ∼1 U/mg) than under low-salt conditions (2.7 U/mg) as previously reported [19]. In striking contrast, we could not detect any activity (<0.001 U/mg) in case of deletion variant TKTΔ38, even when using protein concentrations of up to 2 mg/mL in the assay mixture. At this point, we cannot discriminate whether the lack of activity of TKTΔ38 results from the missing catalytic contributions of the amino acid residues belonging to the deleted 38 mer sequence, or alternatively, from an altered binding of the thiamin cofactor.

### Cofactor Binding Analysis by Near-UV CD and NMR Spectroscopy

Cofactor binding of TKTΔ38 and TKT were analyzed by both near-UV CD and NMR spectroscopy. Previous spectroscopic studies on TKTs and on other proteins of the thiamin enzyme superfamily have revealed that the enzyme-bound cofactor gives rise to prominent near-UV CD bands, which pertain to the different ionization and tautomeric states of the cofactor aminopyrimidine [29], [30].

**Figure 6:**
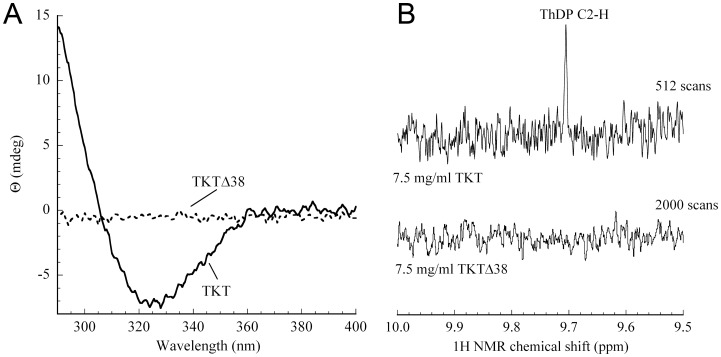
Analysis of cofactor binding in full-length TKT and variant TKTΔ38 by near-UV CD spectroscopy and 1H NMR spectroscopy. (A) Near-UV CD spectra of 2 mg/ml protein in 50 mM glycyl-glycine buffer, pH 7.6 and 500 mM NaCl. Note the absence of the negative CD signal at around 320 nm in case of TKTΔ38, which indicates an impaired binding of the thiamin cofactor in the deletion variant. (B) 1H NMR spectroscopic analysis of supernatant obtained after acid quench treatment of as-isolated TKTΔ38 and full-length TKT. A down-field section of the NMR spectrum (10.0–9.5 ppm) is shown, where the signal of C2-H of the cofactor thiazolium appears. We were unable to detect even traces of the thiamin cofactor in case of TKTΔ38 in contrast to full-length TKT that contains tightly bound ThDP.

In all hitherto characterized TKTs, holo-enzyme formation is associated with build-up of a negative CD band centered around 320 nm [26], [27]. In fact, we do observe the expected negative CD signal at ∼320 nm in case of as-isolated full-length TKT that contains tightly bound ThDP (Fig. 6a). Conversely, no such band can be detected for TKTΔ38 under identical conditions in the as-isolated form as well as after adding excess concentration of cofactors (up to 10 mM thiamin diphosphate and Ca^2+^). This intriguing observation suggests that TKTΔ38 is either unable to bind the thiamin cofactor at all, or, alternatively, that the chemical state of bound cofactor is different than that of other TKTs.

In an independent approach, we tested whether as-isolated TKTΔ38 contains tightly bound thiamin cofactor as recently demonstrated for full-length TKT [19]. In brief, treatment of highly purified protein with an acidic solution leads to complete protein denaturation and release of the cofactor, which can be then quantitatively analyzed by 1H NMR spectroscopy (Fig. 6b) [28]. Our NMR spectroscopic analysis of the supernatant obtained after acid quench treatment of TKTΔ38 unambiguously demonstrates that the as-isolated deletion variant contains no cofactor (<5% active sites). Taken together, the near-UV CD spectroscopic and 1H NMR spectroscopic analysis strongly suggest that TKTΔ38 does not bind the thiamin cofactor in a specific manner.

### Analytical Gel Filtration

The lack of enzymatic activity of TKTΔ38 and its inability to bind the thiamin cofactor raise the question whether the deletion variant may still form the functional homodimeric assembly.

**Figure 7:**
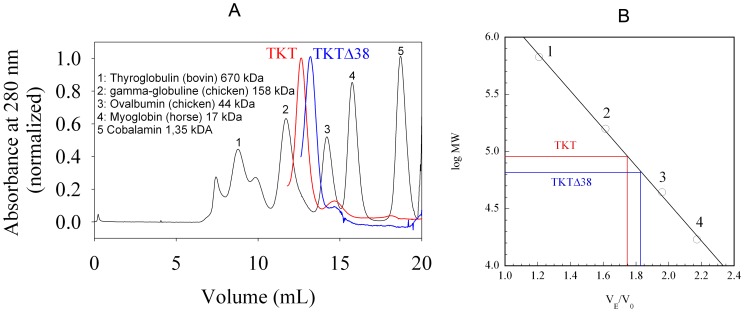
Analytical gel filtration studies on TKTΔ38 and full-length TKT. (**A**) Individual elution profiles (absorbance at 280 nm) of a protein standard mixture, TKT and TKTΔ38. (**B**) Plot of log MW (molecular weight) versus V_E_/V_0_ (V_E_, elution volume; V_0_, void volume). Full conditions are detailed in the Experimental Procedures.

In order to semiquantitatively analyze the oligomeric state of TKTΔ38 and full-length TKT, we carried out analytical gel filtration experiments (Fig. 7). Both proteins elute as a single peak suggesting the presence of one predominant oligomeric species in each case. The retention time of TKTΔ38 compared to full-length TKT is markedly longer than expected for the minor difference in the molecular mass of 4 kDa per monomer. While full-length TKT forms the expected homodimeric assembly (theoretical Mr of dimer ∼135 kDa, estimated Mr ∼120 kDa), the gel filtration experiments strongly suggest the TKTΔ38 variant to mostly exist as monomer (theoretical Mr of monomer ∼64 kDa, estimated Mr ∼70 kDa). We would like to emphasize that the employed gel filtration methodology cannot quantitatively assess the distribution between the monomeric and homodimeric state yet the data demonstrate a shift of the monomer-dimer equilibrium towards the monomer in case of the Δ38 deletion variant. This finding highlights the essential role of the deleted sequence for formation of a functional dimer as suggested by the structure (see Fig. 2).

### Conclusions

The human TKT isoform TKTL1 has attracted exceptional attention as it was suggested to play an important enzymatic role in tumor cell metabolism in terms of an oncogenic gain-of-function [12]. Using quantitative real-time reverse transcriptase PCR, an increased expression of TKTL1 mRNA was detected in several tumor cell lines [31]. In addition, immunohistochemical studies seemed to suggest the presence of TKTL1 in different carcinomas as detected by a monoclonal antibody raised against TKTL1 [12]. Moreover, a direct correlation between the expression levels of TKTL1 and patient prognosis was proposed [13], [14], [15]. It has been hypothesized that TKTL1 exhibits an intrinsic enzymatic activity and converts substrate X5P into G3P and a two-carbon unit, suggested as being acetyl-CoA, in a reaction analogous to the phosphoketolase reaction in heterofermentative bacteria (authentic phosphoketolase generates acetyl phosphate as product) [12]. A very minor TKT activity of enriched native TKTL1 was presented as a proof-of-principle for the postulated enzymatic function. The authors did not provide any specific activities, but complete substrate turnover took as long as 24 hours under conditions, where substrates are completely depleted within a couple of minutes when using authentic human TKT [19]. In further challenge of the postulated role of TKTL1 in tumor cell metabolism, two recent studies could not confirm increased TKTL1 mRNA concentrations in different carcinomas and questioned the validity of the aforementioned immunohistochemical data 16,17. These results match our own observations (Schneider & Tittmann, unpublished) as we could not detect increased TKTL1 transcript levels in cell line HCT116 in contrast to data reported by others [12].

In view of the contradictory data and the lack of reliable biochemical data on TKTL1, we have attempted to establish a recombinant expression system for TKTL1. Since recombinant expression in both prokaryotic and eukaryotic systems resulted in the formation of insoluble protein (Fig. S1, S2), we have genetically engineered a “pseudo-TKTL1” variant of human TKT, for which we have established a robust recombinant expression system and standardized assays [19]. Since the major difference in sequence between TKTL1 and TKT is a deletion of 38 consecutive amino acids in the N-terminal domain (Fig. 1), we have constructed a TKTΔ38 deletion variant lacking residues 76–113. After heterologous expression in *E. coli* and purification to homogeneity in the mg scale, we have biochemically and biophysically characterized TKTΔ38 versus full-length TKT regarding structural integrity at different levels and enzymatic activity under fully standardized conditions. Since we obtain active full-length His-tagged TKT with the bacterial expression system, possible post-translational modifications seem to be nonessential for activity.

Our data unambiguously demonstrate that a deletion of the 38 amino acids in question renders human TKT fully inactive highlighting the importance of this sequence for enzymatic activity. Similar conclusions were derived on the basis of modeling studies on authentic TKTL1 [32]. Moreover, TKTΔ38 fails to bind the thiamin cofactor, even at superphysiological concentrations. While the deletion variant still adopts a fold with a defined secondary structure content, its thermal stability is dramatically reduced compared to full-length TKT. We were unable so far to crystallize TKTΔ38 and to solve its structure by X-ray crystallography using a set of different conditions including those, under which we reproducibly obtain crystals of full-length TKT. The deletion further impairs the thermodynamic stability of the homodimeric assembly. The high sequence identity between TKTL1 and TKTΔ38 suggests that the latter is a good model of TKTL1. This prompts us to propose that TKTL1 does not possess enzymatic activity that relies on thiamin cofactor chemistry. In light of our findings, the proposed intrinsically encoded enzymatic role of TKTL1 in tumor cell metabolism seems to be highly unlikely. Our data cannot ultimately rule out, however, that a putative enzymatic activity of TKTL1 can be restored after binding to hitherto unknown proteins or protein complexes, yet such mechanism would have no reported precedent, at least not for any of the TKTs characterized so far. If TKTL1 indeed serves a biological function in tumor cells, its role seems to be of nonenzymatic nature. This hypothesis needs to be corroborated by biochemical and biophysical analysis of authentic TKTL1 as demonstrated here for the TKT-based deletion variant as a viable model of TKTL1.

## Supporting Information

Figure S1 **Representative SDS-PAGE analysis of TKTL1 expression in** ***E. coli*** BL21*. Note that TKTL1 is not expressed in soluble form.
(PDF)

Figure S2 **Representative Western Blot analysis of TKTL1 expression in** ***Trichoplusi ni*** **Hi5 cells.** Note the absence of soluble TKTL1 (supernatant and eluate of Ni-NTA column). Similar results were obtained for TKTL1 expression in Sf9 and Sf21 cells.
(PDF)

Methods S1 Gene expression and purification of human TKTL1 in E. coli BL21*. Gene expression and purification of human TKTL1 in *Spetoptera frugiperda* 9/21 and *Trichoplusi ni* Hi5 cells.
(DOCX)
