# Supplementary material for: A Δ38 Deletion Variant of Human Transketolase as a Model of Transketolase-Like Protein 1 Exhibits No Enzymatic Activity
Source: PLoS One. 2012 Oct 31;7(10):e48321. doi: 10.1371/journal.pone.0048321 (PMC3485151; doi:10.1371/journal.pone.0048321)
Supplement: Figure S1 — Representative SDS-PAGE analysis of TKTL1 expression in E. coli BL21*. Note that TKTL1 is not expressed in soluble form. (PDF) [file pone.0048321.s001.pdf]

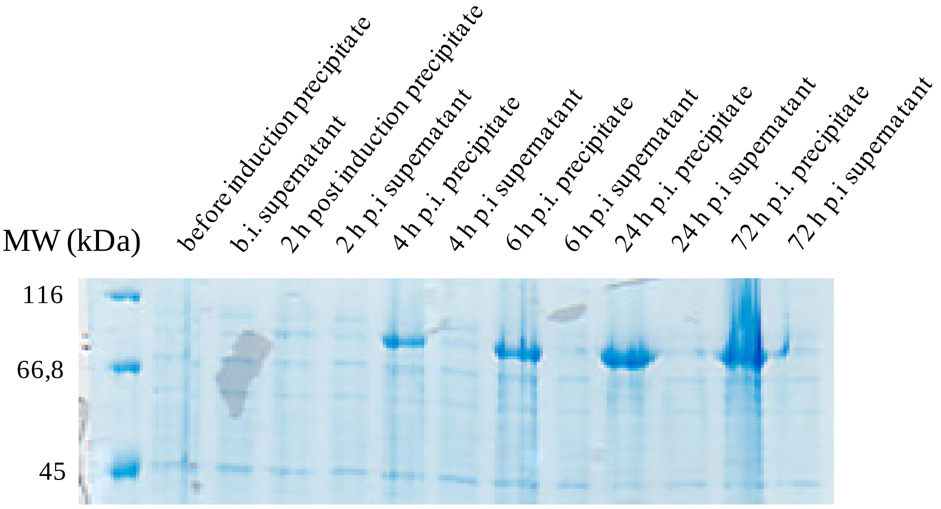

Fig. S1. Representative SDS-PAGE analysis of TKTL1 expression in *E. coli* BL21*. Note that TKTL1 is not expressed in soluble form.
