# Supplementary material for: A Δ38 Deletion Variant of Human Transketolase as a Model of Transketolase-Like Protein 1 Exhibits No Enzymatic Activity
Source: PLoS One. 2012 Oct 31;7(10):e48321. doi: 10.1371/journal.pone.0048321 (PMC3485151; doi:10.1371/journal.pone.0048321)
Supplement: Figure S2 — Representative Western Blot analysis of TKTL1 expression in Trichoplusi ni Hi5 cells. Note the absence of soluble TKTL1 (supernatant and eluate of Ni-NTA column). Similar results were obtained for TKTL1 expression in Sf9 and Sf21 cells. (PDF) [file pone.0048321.s002.pdf]

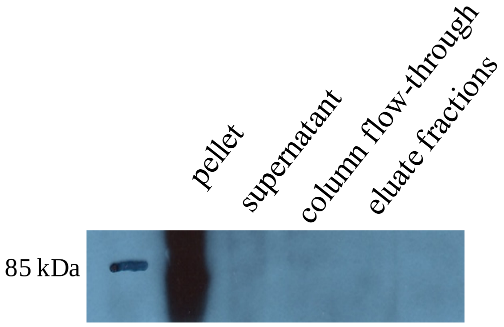

Fig. S2. Representative Western Blot analysis of TKTL1 expression in *Trichoplusi ni* Hi5 cells. Note the absence of soluble TKTL1 (supernatant and eluate of Ni-NTA column). Similar results were obtained for TKTL1 expression in Sf9 and Sf21 cells.
