# Supplementary material for: A Δ38 Deletion Variant of Human Transketolase as a Model of Transketolase-Like Protein 1 Exhibits No Enzymatic Activity
Source: PLoS One. 2012 Oct 31;7(10):e48321. doi: 10.1371/journal.pone.0048321 (PMC3485151; doi:10.1371/journal.pone.0048321)
Supplement: Methods S1 — Gene expression and purification of human TKTL1 in E. coli BL21*. Gene expression and purification of human TKTL1 in Spetoptera frugiperda 9/21 and Trichoplusi ni Hi5 cells. (DOCX) [file pone.0048321.s003.docx]

***Gene expression and purification of human TKTL1 in E.coli BL21****

The pETSUMO vector encoding for C-terminally SUMO-tagged human transketolase-like protein 1 was transformed into *E. coli* BL21* cells (Invitrogen). Transformed cells were grown on LB/Agar plates containing 35 μg/mL kanamycin over night at 37 °C. A single colony was used to inoculate 200 mL of overnight culture (LB medium supplemented with 35 μg/mL kanamycin) in a 1 L Erlenmeyer flask. The overnight culture was centrifuged (6000 rpm, 20 min, 4 °C), and cells were resuspended in 100 mL LB medium containing 35 μg/mL kanamycin. This cell suspension was then used to inoculate 400 mL of LB medium further containing 35 μg/mL kanamycin and 70 μg/mL thiamin. At an OD_600_ of ~0.6, gene expression was induced by addition of 100 μM IPTG, and cell cultures were grown for 18 h at 12 °C and 200 rpm. Cells were harvested by centrifugation at 6000*g* for 20 min at 4 °C. Thereafter, harvested cells were shock-frozen in liquid nitrogen and stored at -80 °C until usage. For cell lysis, 5 g of cells were thawed and resuspended in 20 mL of buffer containing 100 mM Tris-HCl pH 8.0, 1.5 M NaCl, 100 mM imidazole, 5 mM CaCl_2_ and 300 µM thiamin diphosphate. Moreover, DNase (5 μg/mL), MgCl_2_ (1 mM), lysozyme (0.2 mg/mL) and PMSF (1 mM) were added. The suspension was stirred on ice for 30 min. Cells were disrupted by sonification (three times, each for a 30 s duration) on ice using a sonifier cell disruptor B15 (Branson Sonic Power), and this extract was centrifuged at 15000*g* for 15 min at 4 °C to clear debris, yielding crude bacterial extract. Precipitate (insoluble) and supernatant (soluble) fractions were analyzed for TKTL1 expression by SDS-PAGE (Fig. S1).

***Gene expression and purification of human TKTL1 in Spetoptera frugiperda 9/21 and Trichoplusi ni Hi5 cells.***

DNA encoding for human transketolase-like protein 1 was transferred as NcoI/XhoI amplicons into transfer vector pFastBacHTb. Baculovirus DNA was generated by the Bac-to-Bac TOPO expression system according to the manufacturer’s manual (Invitrogen). The three used cell lines (*Spetoptera frugiperda* 9 and 21, *Trichoplusi ni* Hi5) were infected with a P3 stock of the corresponding viruses (titer determined before infection) to induce protein expression. Protein expression was obtained after 24 to 48 hours.

For protein purification, 5 g of the corresponding cells were thawed and resuspended in 20 mL of buffer containing 20 mM Tris-HCl pH 8.0, 300 mM NaCl, 10 mM imidazole, 1 mM CaCl_2_ and 100 µM thiamin diphosphate. Moreover, DNase (5 μg/mL), MgCl_2_ (1 mM), lysozyme (0.2 mg/mL) and PMSF (1 mM) were added. The suspension was stirred on ice for 30 min. Cells were disrupted by sonification (three times, each for a 30 s duration) on ice using a sonifier cell disruptor B15 (Branson Sonic Power), and this extract was centrifuged at 50000*g* at 4 °C for 30 min to clear debris yielding crude extract. The supernatant was loaded onto a Ni^2+^-NTA column (GE Healthcare) previously equilibrated with 10 column volumes of 20 mM Tris-HCl, pH 8.0, 300 mM NaCl, 10 mM imidazole, 1 mM CaCl_2_ and 100 μM thiamin diphosphate. TKTL1 was eluted using a linear gradient (0-100%) elution buffer (20 mM Tris-HCl, pH 8.0, 300 mM NaCl, 1 mM CaCl_2_, 100 μM thiamin diphosphate and 200 mM imidazole) (Fig. S2). TKTL1 expression was tested by Western Blot analysis (antibody against N-terminal His-tag).
